# Effects of Adenosine on Lymphangiogenesis

**DOI:** 10.1371/journal.pone.0092715

**Published:** 2014-03-20

**Authors:** Bénédicte Lenoir, Daniel R. Wagner, Silvia Blacher, Graciela B. Sala-Newby, Andrew C. Newby, Agnès Noel, Yvan Devaux

**Affiliations:** 1 Laboratory of Cardiovascular Research, Centre de Recherche Public de la Santé (CRP – Santé), Luxembourg; 2 Division of Cardiology, Centre Hospitalier Luxembourg, Luxembourg; 3 Laboratory of Tumor and Development Biology, Groupe Interdisciplinaire de Génoprotéomique Appliquée - Cancer, University of Liège, Liège, Belgium; 4 Bristol Heart Institute, Bristol Royal Infirmary, University of Bristol, Bristol, United Kingdom; I2MC INSERM UMR U1048, France

## Abstract

**Background:**

The lymphatic system controls tissue homeostasis by draining protein-rich lymph to the vascular system. Lymphangiogenesis, the formation of lymphatic vessels, is a normal event in childhood but promotes tumor spread and metastasis during adulthood. Blocking lymphangiogenesis may therefore be of therapeutic interest. Production of adenosine is enhanced in the tumor environment and contributes to tumor progression through stimulation of angiogenesis. In this study, we determined whether adenosine affects lymphangiogenesis.

**Methods:**

Lymphatic endothelial cells (HMVEC-dLy) were cultured in presence of adenosine and their proliferation, migration and tube formation was assessed. Gelatin sponges embedded with the stable analogue of adenosine 2-chloro adenosine were implanted in mice ear and lymphangiogenesis was quantified. Mice were intravenously injected with adenoviruses containing expression vector for 5′-endonucleotidase, which plays a major role in the formation of adenosine.

**Results:**

In vitro, we observed that adenosine decreased the proliferation of lymphatic endothelial cells, their migration and tube formation. However, in vivo, gelatin sponges containing 2-chloro adenosine and implanted in mice ear displayed an elevated level of lymphangiogenesis (2.5-fold, p<0.001). Adenovirus-mediated over-expression of cytosolic 5′-nucleotidase IA stimulated lymphangiogenesis and the recruitment of macrophages in mouse liver. Proliferation of lymphatic endothelial cells was enhanced (2-fold, p<0.001) when incubated in the presence of conditioned medium from murine macrophages.

**Conclusion:**

We have shown that adenosine stimulates lymphangiogenesis in vivo, presumably through a macrophage-mediated mechanism. This observation suggests that blockade of adenosine receptors may help in anti-cancer therapies.

## Introduction

Lymphatic vessels are found all over the human body except in certain tissues or organs such as epidermis, cartilage, brain, cornea, bone marrow and retina. The lymphatic system controls the homeostasis of tissue fluid by draining protein-rich lymph from the tissues and organs back to the vascular system. It also contributes to intestinal lipid absorption and to the transport of lymphocytes and dendritic cells [1], [2]. A deleterious role of the lymphatic system has been evidenced in cancer, in which lymphatic vessels participate in the promotion of tumor growth and metastasis [3]. Also, dysfunction of lymphatic vessels in tumors can reduce the efficacy of anti-cancer drugs [4], [5].

Lymphangiogenesis is the formation of new lymphatic vessels, a normal event in childhood. During adulthood, lymphangiogenesis is associated with pathological conditions such as inflammation, healing, graft rejection, auto-immune diseases and tumor progression [1], [2], [4], [6]. Through the secretion of growth factors and pro-lymphangiogenic cytokines, inflammatory cells stimulate lymphangiogenesis [2], [6]–[8]. Macrophages are the main actors of inflammatory lymphangiogenesis [9], principally through the secretion of vascular endothelial growth factor-C (VEGF-C) and VEGF-D [2], [7]. Other paracrine factors secreted by macrophages also share pro-lymphangiogenic properties which drive the growth, morphogenesis and function of lymphatic endothelial cells (LEC) [10]–[12]. In addition, a subset of macrophages which possesses the ability to transdifferentiate into LEC have been termed macrophage-derived lymphatic progenitors (M-LEC) [1], [2], [10], [13]. These macrophages co-express the macrophage marker F4/80 and the lymphatic marker podoplanin [11].

Modulation of lymphangiogenesis is expected to have some therapeutic value in certain pathological conditions such as lymphedema, tumor metastasis, Kaposi sarcoma and obesity [14]. Blocking antibodies against VEGF receptor -3 (VEGFR-3), the receptor of pro-lymphangiogenic VEGF-C, have recently entered clinical trials as anti-angiogenic and anti-metastatic drugs [15]. However, there is still a paucity of clinically applicable tools to modulate lymphangiogenesis.

Adenosine is an ubiquitous and endogenous purine nucleoside with a plethora of physiological functions [16]. Although constitutive, the secretion of adenosine is increased under metabolic stress such as hypoxia, inflammation and cancer. In addition to its extensively documented role in inflammation, adenosine has been shown to be a master regulator of angiogenesis [17]–[21]. However, whether adenosine affects lymphangiogenesis is unknown.

Adenosine binds to 4 types of receptors (A1, A2a, A2b and A3), all belonging to class A family of G-protein coupled receptor family [22]. Several pharmacological agents have been developed to specifically activate or inhibit adenosine receptors. Thus, adenosine receptors are appealing therapeutic targets [23].

In the present study, we hypothesized that adenosine may regulate lymphangiogenesis. Using different in vitro and in vivo models [24], we provide evidence that adenosine inhibits the proliferation and migration of LEC in vitro but stimulates lymphangiogenesis in vivo.

## Materials and Methods

### *In vitro* experiments

#### Cell culture

Human adult dermal microvascular lymphatic endothelial cells (LEC) were purchased from Lonza (HMVEC-dLy; Braine-l′Alleud, Belgium) and used at passages 3 to 5. Cells were cultured at 37°C in a 5% CO_2_ atmosphere in endothelial growth microvascular (EGM2-MV) medium (Lonza) composed of EBM2 medium with 5% FBS, hydrocortisone, h-FGF-B, VEGF, R3-IGF-1, ascorbic acid, hEGF and GA 1000 [9]. For drug treatments, cells were washed and cultured in EGM2-MV medium supplemented with 2% FBS, and half of the medium was renewed every 24 h. Adenosine (Sigma-Aldrich) was used at concentration ranging from 0.1 μM to 10 μM and the adenosine deaminase inhibitor EHNA (Sigma) was used at a concentration of 10 μM to increase adenosine half-life. CGS21680 and NECA (Sigma-Aldrich) were used as preferential A2a and A2b agonists, respectively.

Primary macrophages were isolated from peritoneal lavage of C57BL/6 mice intraperitoneally injected with 4% thioglycollate (Sigma-Aldrich, St. Louis, MO) 5 days earlier. After washing off non-adherent cells, macrophages were cultured in serum-free medium (RPMI-1640, Lonza) and conditioned medium was collected.

#### Viability/cytotoxicity assay

Drug toxicity was assessed with the live/dead viability/cytotoxicity kit (Molecular Probes, Invitrogen) according to the manufacturer's protocol. Six technical replicates per test were performed.

#### Proliferation assay

LEC (4×10^3^) cultured in EBM2-MV medium containing 2% FBS were treated with adenosine or with 50% conditioned medium from macrophages treated by adenosine. Half of the medium was changed every 24 hours. Cell proliferation was assessed using CyQUANT proliferation kit (Lonza).

#### Boyden chamber assay

To study the migration of LEC, we used Boyden chambers (Corning Inc., Corning, Amsterdam) with filters of 8 μm pore size previously coated with 0.005% gelatin (Type A, porcine skin, Sigma). Fifty thousand LEC were seeded in the upper chamber, in EBM2-MV containing 2% FBS. Adenosine at concentrations ranging from 0.1 μM to 10 μM was added in the lower chamber. To study the migration of macrophages, murine peritoneal macrophages were deposited on 5 μm pore size in RPMI medium and incubated in presence of adenosine and EHNA (10 μM). After 24 h for LEC and 4 h for macrophages, cells in the upper chamber were carefully removed using cotton buds and cells at the bottom of the membrane were fixed and stained with 4% Giemsa. Quantification was performed by counting the stained cells on the membrane. All assays included 3 technical replicates.

#### Wound healing assay

LEC (3×10^4^) were cultured in EGM2-MV medium in a culture insert (Ibidi, Proxylab, Belgium). When cells reached confluence, the insert was removed and the wounded monolayers were washed with serum-free medium. Cells were then treated with different concentrations of adenosine in EBM2 containing 2% FBS. Culture plates were placed in a live cell imaging platform (Cell IQ, Chip-man technologies, UK) and 3 pictures per well were acquired automatically each 30 min according to fixed reference points. The width of the wound was determined at time 0 and 8 h, 16 h and 24 h after experiment onset. All assays included 3 technical replicates.

#### Tubulogenesis assay

LEC (8×10^5^) were seeded on a type I collagen layer (1 mg/ml, Collagen R; Serva Electrophoresis, Heidelberg, Germany) in a 6-well plate. Cells were grown for 24 hours in complete EGM2-MV medium. Medium was then removed and a second layer of collagen was added over the cells. Finally, collagen-embedded were incubated in 2 mL of EGM2-MV medium containing or not adenosine. After 24 hours, 5 pictures per well were captured with a phase-contrast microscope (Axiovert 25; Carl Zeiss) coupled to an Axiocam color digital camera (Carl Zeiss) and tube length was measured [9].

#### Spheroid assay

LEC (1.5×10^3^ cells per well in 24 well plates) were pre-cultured for 24 h in EBM-2 containing 0.24% high viscosity methyl cellulose (Sigma-Aldrich) to form microspheres. Subsequently, spheroids were collected, embedded in collagen gels with 100 ng/ml PMA and maintained at 37°C for 24 h in EBM2-MV medium including 2% FBS, with or without adenosine. Spheroids were examined by phase-contrast microscopy using an Axiovert 25 microscope equipped with a 20 NA 0.3 LD A-Plan lens and an Axiocam color digital camera (Carl Zeiss). Images were captured at room temperature, using acquisition software KS400 3.0. Cell migration was quantified by a computerized method determining the sprouting envelope area, defined as the area of the minimal convex polygon containing the whole spheroid and all sprouting cells.

#### Real-time quantitative PCR

Total RNA from cultured cells was isolated using TriReagent and the RNeasy mini kit (Qiagen, Venlo, Netherlands). Potential contaminating genomic DNA was digested by DNase I treatment (Qiagen). One microgram of total RNA was reverse-transcribed using the Superscript® II Reverse Transcriptase (Invitrogen, Merelbeke, Belgium). PCR primers were designed using the Beacon Designer software (Premier Biosoft, Palo Alto, USA) and were chosen to encompass an intron. PCR was performed using the iCycler and the IQ SYBR Green Supermix (Biorad, Nazareth, Belgium). PCR conditions were as follows: 3 min at 95°C, 30 s at 95°C and 1 min annealing (40-fold). Optimal annealing temperature was determined for each primer pair (Table 1). Melting point analysis was obtained after 80 cycles of 10 s from 55°C to 95°C. Each run included negative reaction controls. β-actin was chosen as housekeeping gene for normalization. Expression levels were calculated by the relative quantification method (ΔΔCt) using the Genex software (Biorad) which takes into account primer pair efficiency.

**Table 1:** Quantitative PCR primers.

Gene	Primer sequence	Annealing temperature	Amplicon length
ADORA1	Sense: 5′-GACCTACTTCCACACCTG-3′ Anti-sense: 5′-TCACCACCATCTTGTACC-3′	58°C	140 bp
ADORA2A	Sense: 5′-TCTTCAGTCTCCTGGCCATC-3′ Anti-sense: 5′-GGGACCACATCCTCAAAGAG-3′	64°C	244 bp
ADORA2B	Sense: 5′-CTCCATCTTCAGCCTTCTGG-3′ Anti-sense: 5′-ACAAGGCAGCAGCTTTCATT-3′	58°C	234 bp
ADORA3	Sense: 5′-TCATCTGCGTGGTCAAGC-3′ Anti-sense: 5′-CTGTAGAAGTGGATTGTGATGC-3′	62°C	148 bp
β-actin	Sense: 5′-AGAAAATCTGGCACCACACC -3′ Anti-sense: 5′-GGGGTGTTGAAGGTCTCAAA-3′	60°C	142 bp

### In vivo experiments

#### Mice

Ten-week-old male C57BL/6 mice purchased from Janvier (Le Genest St Isle, France) were used throughout this study. Animal experiments were performed in compliance with the local Animal Ethical Committee of the University of Liege (Liege, Belgium) who specifically approved this study.

#### Collagen lymphangiogenesis assay

Gelatin sponges (Gelfoam, Pfizer, Puurs, Belgium) were cut in small scares of approximately 0.5 cm^2^. After incubation in 20 μl CADO (0.003 mg/ml) (2-chloroadenosine) (Sigma), recombinant VEGF-C (1 ng/ml) (R&D System, Oxon Abingdon, UK) or MRS1754 (0.2 mg/kg) (2.4 mg/ml) (Sigma) with or without CADO, sponges were embedded in interstitial type I collagen gel (1.5 mg/mL; Serva). Then sponges were implanted between the two skin layers of mice ear. After 3 weeks, tissues were excised and sponges were embedded and frozen in optimal cutting temperature (OCT) compound. Immunofluorescent staining for mouse LYVE-1 (R&D System) was performed using a secondary antibody labeled with Alexa-Fluor 488 (Molecular Probes, Invitrogen). Cell nuclei were counterstained with Dapi Fluoromount G (Southern Biotech). Slices were scanned by Nanozoomer (Hamamatsu, Mont-Saint-Guitbert, Belgium). A computer-assisted method of quantification was used to determine the number of vessels per mm^2^. Each experimental group contained 3 mice.

#### Adenoviral-mediated over-expression of cytosolic 5′-nucleotidase IA in mice

We used two adenoviral vectors prepared at the Bristol Heart Institute (Bristol Royal Infirmary, University of Bristol, United Kingdom) as previously described [25]: a control vector containing a GFP cDNA as tag (Ad-GFP vector) and a vector containing the coding sequence of pigeon cN-IA (5′-nucleotidase) without GFP cDNA (Ad-cN-IA vector). Virus stocks were amplified, CsCl banded and titrated before use. Vectors (Ad-cN-IA or Ad-GFP) (10^9^ pfu/animal) or PBS were injected through the tail vein. Mice were sacrificed 2 weeks later. Liver was excised, fixed in 4% paraformaldehyde and embedded in paraffin. Immunohistochemical stainings were performed for the lymphatic vessel marker podoplanin (goat anti-mouse podoplanin antibody, R&D System), the pan-leukocyte marker CD45 (rat anti-mouse CD45 antibody, BD Pharmingen, BD Bioscience, Belgium), and the macrophage marker F4/80 (rat anti-mouse F4/80 antibody, AbD Serotec, Biorad, Düsseldorf, Germany). For CD45 and F4/80 immunostainings, secondary antibodies coupled to streptavidin/HRP (DAKO) were used. For podoplanin, a tertiary antibody coupled to streptavidin/HRP (DAKO) was used. A rabbit polyclonal antibody [26] was used for cN-I immunostaining. Slices were counterstained with hematoxylin-eosin and scanned by Nanozoomer (Hamamatsu). Image processing and signal quantification were performed using Aphelion 3.2 software (Adcis, Saint-Contest, France) and image analysis toolbox of Matlab 7.9 software (The Mathworks, Inc., Natick, MA).

## Results

### Lymphatic endothelial cells express adenosine receptors

First of all, we determined the expression profile of adenosine receptors in human adult dermal microvascular lymphatic endothelial cells (HMVEC-dLy = LEC) using quantitative PCR. Lymphatic endothelial cells expressed the A2a and A2b adenosine receptors, but not the A1 and A3 subtypes. Having verified that LEC express adenosine receptors, we investigated the effect of adenosine on different biological functions of these cells. In these experiments performed in vitro, LEC were treated with various concentrations of adenosine along with 10 μM of the adenosine deaminase inhibitor EHNA. This drug slows down the degradation of adenosine into inosine, thereby sustaining the effects of adenosine which has a very short half-life. This strategy has been previously used and discussed [27]–[29].

### Adenosine decreases the proliferation of LEC

**Figure 1:**
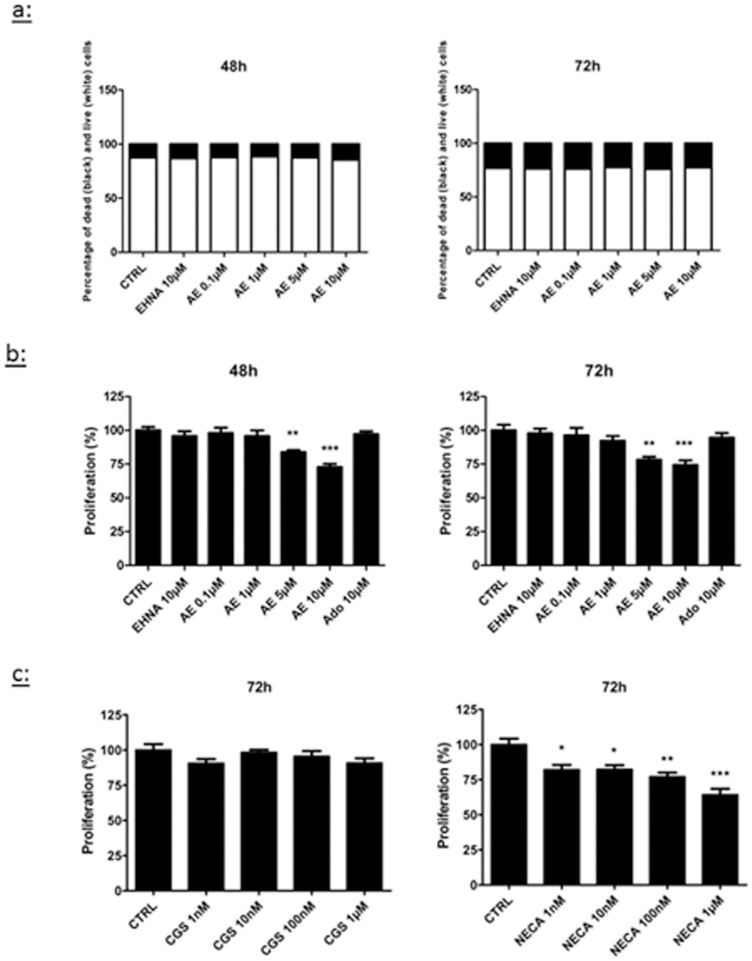
Effect of adenosine on LEC viability and proliferation. Cell viability (a) and proliferation (b–c) were evaluated in HMVEC-dLy ( = LEC) cultured for 48 h (left panel) or 72 h (right panel) in medium containing 2% FBS (CTRL). Cells were treated with EHNA alone (EHNA 10 μM), EHNA with different concentrations of adenosine (AE), the A2a agonist CGS21680 or the A2b agonist NECA. For cell viability (a), results are expressed as percentage of dead cells (black) and living cells (white). Cell proliferation (b–c) was measured with a CyQANT assay. * p<0.05 vs control (CTRL), *** p<0.001 vs CTRL. Data are expressed as mean ± SEM (n = 3).

First, we tested whether adenosine modulated LEC proliferation. Initial experiments were performed to address the toxicity of adenosine and EHNA. Using the live/dead viability/cytotoxicity assay, we observed that a combined treatment with adenosine and EHNA did not affect cell viability for concentrations up to 10 μM (Fig.1a). Then, we evaluated the proliferation of LEC with the CyQUANT proliferation assay and we observed that treatments with 5 μM and 10 μM adenosine for 48 h and 72 h decreased the proliferation rate (Fig.1b). This decrease reached 30% with 10 μM adenosine after 72 h. Of note, no effect was observed before 48 h (not shown). The inhibition of proliferation induced by adenosine was reproduced by the A2b agonist NECA, but not by the A2a agonist CGS21680 (Fig.1c).

### Adenosine decreases the migration of LEC

**Figure 2:**
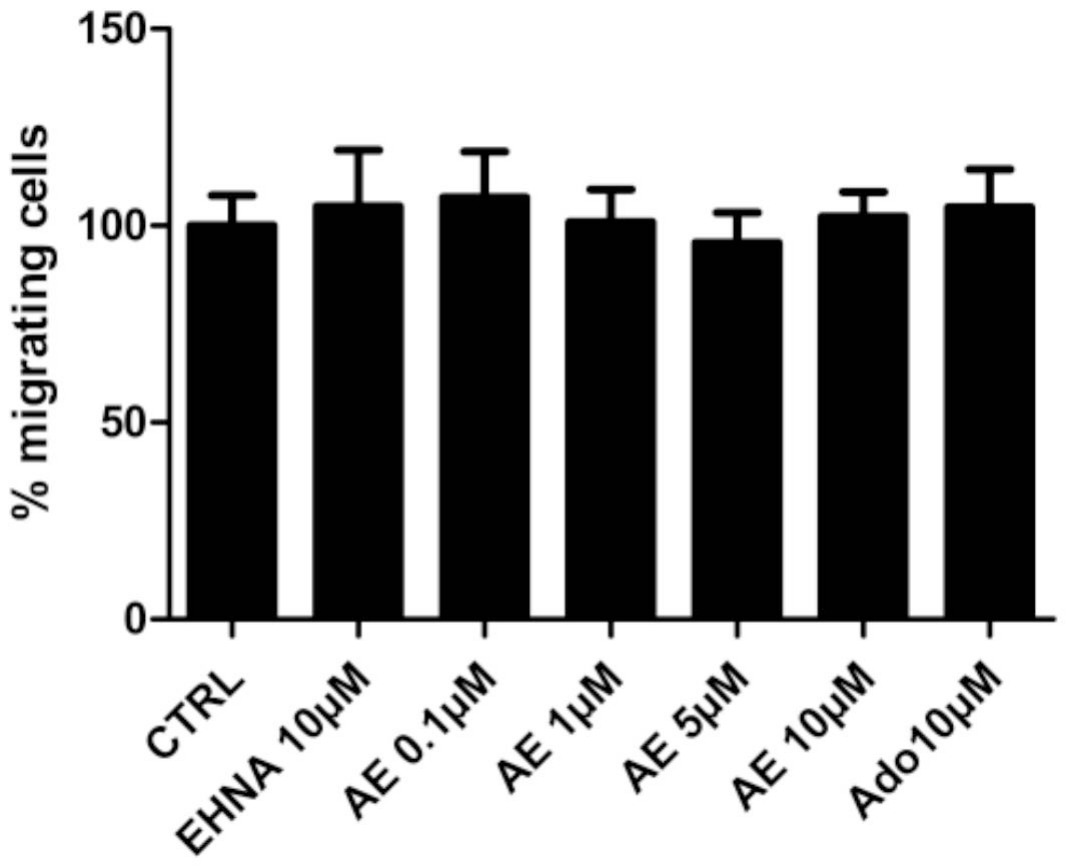
Effect of adenosine on the migration of LEC in Boyden chamber. HMVEC-dLy were cultured in medium containing 2% FBS (control condition, CTRL), with or without 10 μM EHNA and 0.1–10 μM adenosine (AE). Cell migration was assessed in a Boyden chamber assay 24 hours after treatment onset. There was no difference on cell migration between all treatments. Results are expressed as percentage of migrating cells (mean ± SEM, n = 3).

**Figure 3:**
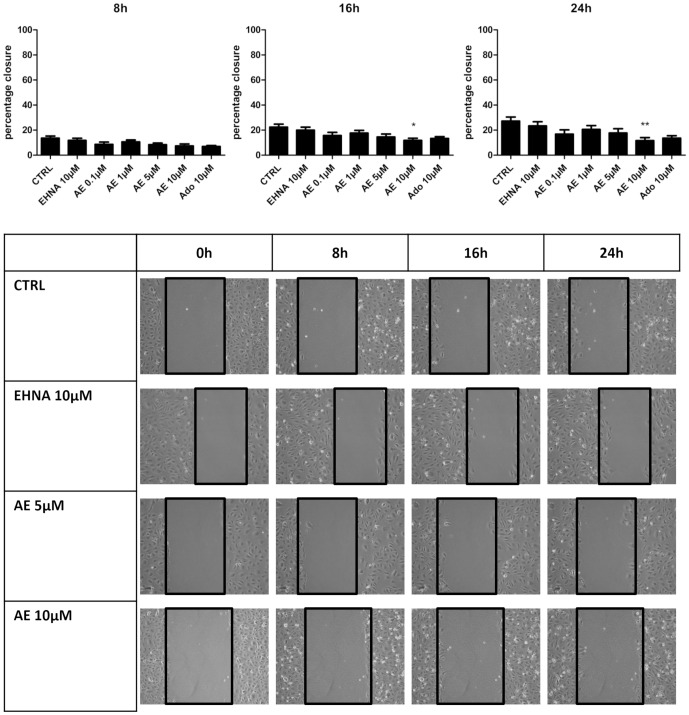
Effect of adenosine on LEC migration in scratch test. HMVEC-dLy were treated during 24 h in medium containing 2% FBS, in the presence of EHNA alone (EHNA, 10 μM) or EHNA with different concentrations of adenosine (AE). Pictures were taken at 0 h, 8 h, 16 h and 24 h after insert removing. Results are expressed as percentage of wound closure (percentage closure) (mean ± SEM). * p<0.05 vs CTRL, ***p<0.001 vs CTRL. Each experiment was performed three times and a representative picture of each condition is shown.

The effect of adenosine on the migration of LEC was evaluated using two different methods. Firstly, we used a Boyden chamber assay in which adenosine placed in the bottom chamber was used as a chemoattractant. Adenosine did not affect the migration of LEC (Fig.2). Secondly, we used a wound healing assay with inserts allowing to standardize the width of the scar. As shown in Fig. 3, the scar was closed by 30% after 24 h, and this closure was inhibited when cells were treated with 10 μM adenosine. EHNA alone had no effect but it's co-administration with adenosine was necessary to observe a significant effect. Collectively, these data show that adenosine itself is not a chemoattractant of LEC and inhibits their migration.

### Adenosine decreases tube formation from lymphatic endothelial cells

**Figure 4:**
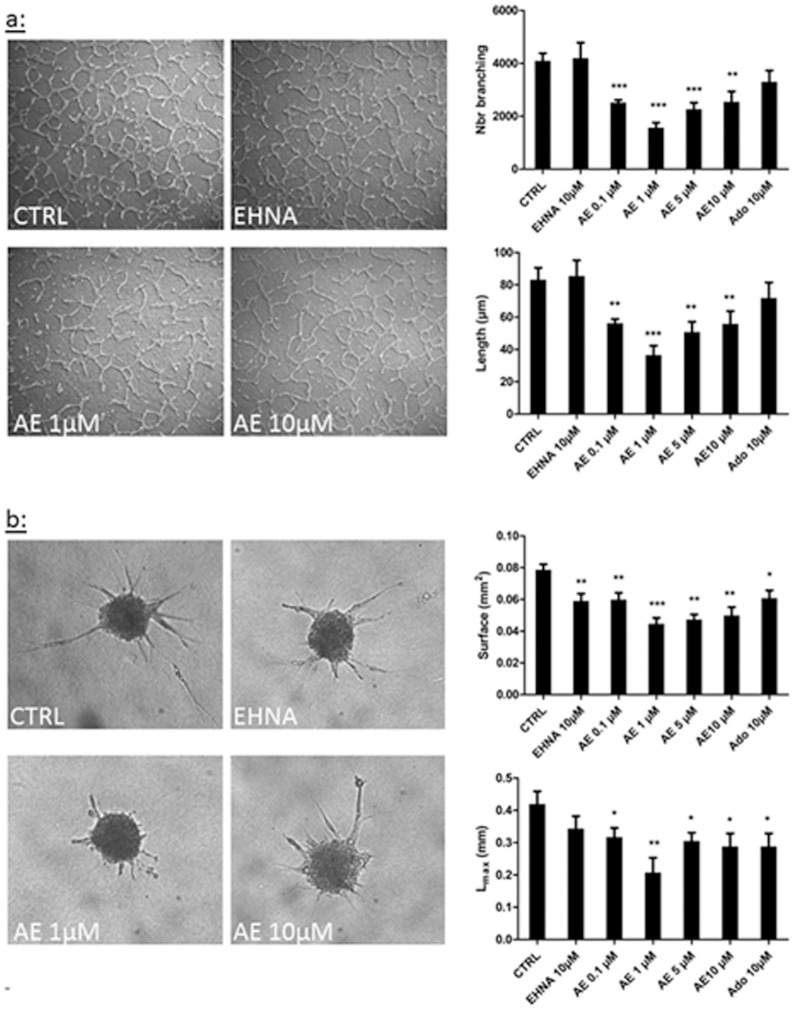
Effect of adenosine on LEC tube formation. Tube formation was assessed in two in vitro models, the tubulogenesis assay (a) and the spheroid assay (b). HMVEC-dLy were cultured in 2% FBS medium (CTRL) and treated or not with EHNA alone (EHNA 10 μM) or EHNA with different concentrations of adenosine (AE). Quantification of tube formation (a) and cell migration (b) was performed by a computerized method on pictures taken after 24 h of culture. The parameters measured are: the tubes branching (branching), the length of tube (length), the surface occupied by tube (surface), and the maximal length of tube (Lmax). * p<0.05 vs CTRL, **p<0.01 vs CTRL, *** p<0.001 vs CTRL. Each experiment was performed three times and representative pictures are shown. Data are expressed as mean ± SEM.

The impact of adenosine on the differentiation of LEC into tube-like structures was investigated using two models. First, we used a tubulogenesis assay in which cells were embedded between two collagen layers. After 24 h, a network of tube-like structures emanating from the LEC was observed in control condition (Fig.4a). When cells were treated with adenosine however, the vascular network was disorganized, and its surface and the maximal length of the tubes were decreased. The effect was maximal with 1 μM of adenosine (2-fold decrease). Second, we used a spheroid assay in which micro-spheres of LEC were embedded in a collagen gel. After 24 h, a network of tube-like structures was visible in the control condition (Fig.4b). Adenosine pre-treatment resulted in a reduced LEC outgrowth. Again, the effect was maximal with 1 μM adenosine (2-fold decrease). EHNA or adenosine alone had not effect. Together, these experiments show that adenosine inhibits lymphangiogenesis in vitro.

### A stable analog of adenosine increases lymphangiogenesis in gelatin sponges in vivo

**Figure 5:**
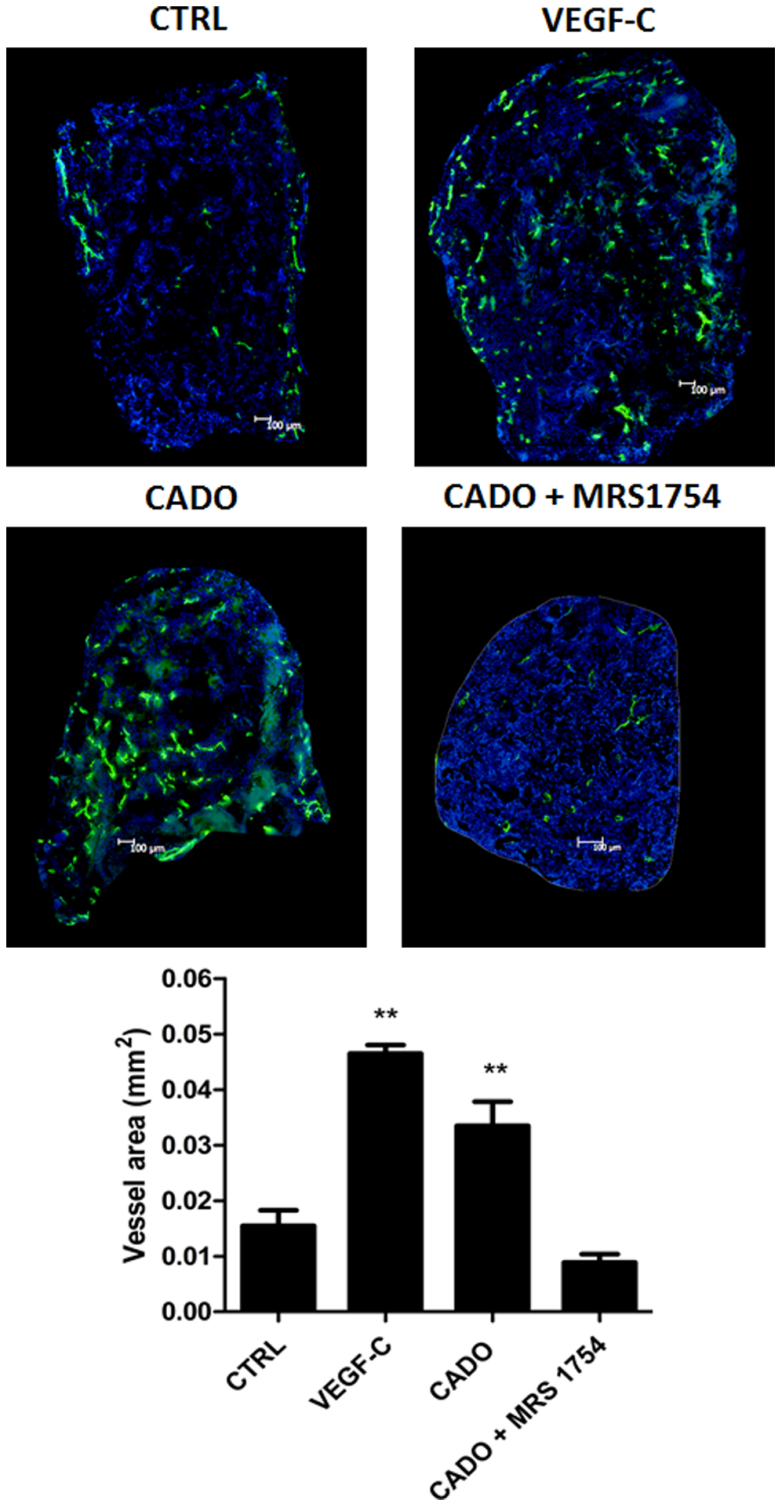
Effects of adenosine on lymphangiogenesis in the in vivo model of collagen sponge. Sponges were soaked with PBS as control (CTRL), with VEGF-C (1 ng/ml) as positive control (VEGF-C), with 20 μl CADO (3 ng/ml), a stable analog of adenosine, or with CADO in presence of the A2b antagonist MRS1754 (2.4 mg/ml). Sponges were implanted between the two skin's layers of ear's mice for 3 weeks. Every other day, PBS or MRS1754 were injected in the apex of the ear. Sponge sections were stained with an anti-Lyve-1 antibody to detect lymphatic vessels (green) and Dapi to detect cell nucleus (blue). The graph corresponds to computerized quantification of the surface occupied by lymphatics (vessel area). Data are expressed as mean ± SEM (n = 6). ** p<0.01 vs CTRL.

Following in vitro experiments, we sought to determine whether adenosine affects lymphangiogenesis in vivo. We first used a collagen lymphangiogenesis assay with gelatin sponges. Sponges were embedded in collagen containing either the stable analogue of adenosine 2-chloro adenosine (CADO), VEGF-C as a positive control, or the A2b receptor antagonist MRS1754. Sponges were implanted between the two skin layers of mice ear. PBS or MRS1754 were injected at the apex of the ear every two days. After 3 weeks, sponges were removed, frozen and sliced for immunostaining for LYVE-1 lymphatic marker. As expected, sponges containing VEGF-C displayed a higher level of lymphangiogenesis compared to control condition, as assessed by the area of the sponges occupied by lymphatic vessels (Fig.5). Interestingly, a 2.5-fold increase in lymphatic vessels was observed in CADO-containing sponges, as compared to control. This increase was blunted by MRS1754. Of note, administration of MRS1754 alone did not affect lymphangiogenesis (not shown). These results show that the stable analog of adenosine CADO is able to stimulate lymphangiogenesis in vivo, presumably through the adenosine A2b receptor.

### Over-expression of 5′-nucleotidase increases lymphangiogenesis in the murine liver

**Figure 6:**
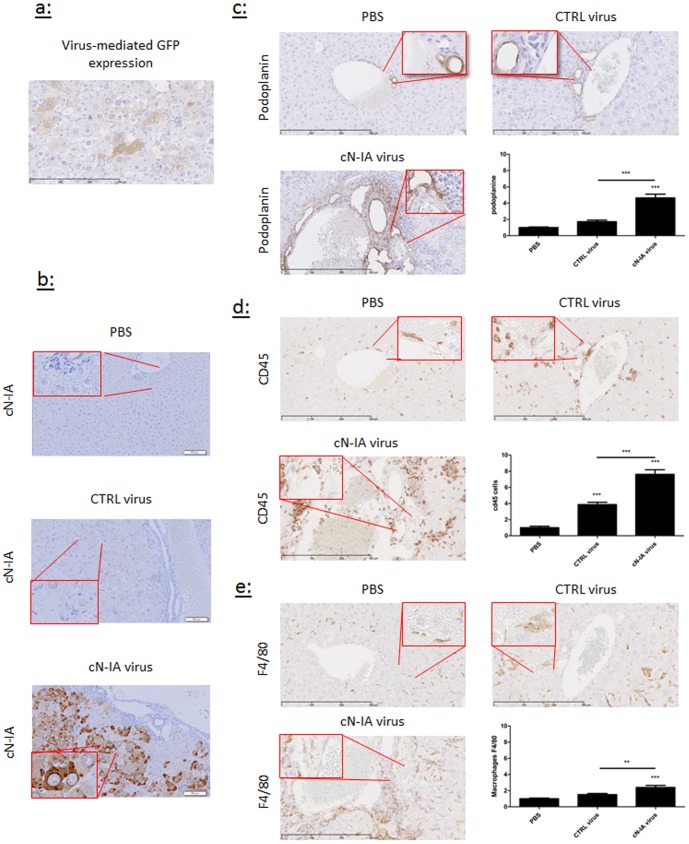
Effect of adenosine on lymphatic vasculature and inflammatory cell recruitment in the liver. Control adenovirus expressing GFP (CTRL virus) or adenovirus carrying the sequence of 5-nucleotidase (cN-IA virus) were injected in the caudal vein of mice. PBS was used as negative control (PBS). Mice were sacrificed 2 weeks after injection. The efficacy of virus transduction was shown by GFP immunostaining (a) and by cN-IA immunostaining (b)). Liver sections were stained with anti-podoplanin antibodies to detect lymphatic vessels (c), with anti-CD45 antibodies to detect inflammatory cells (d), and with anti-F4/80 antibodies to detect macrophages (e). Data are presented as mean ± SEM (n = 15 for PBS, n = 13 for GFP and n = 12 for cN-IA).**p<0.01, *** p<0.001. Representative pictures are shown.

To confirm the effect of adenosine on lymphangiogenesis observed in gelatin sponges, we used an adenovirus overexpressing 5′-nucleotidase (cN-IA), an isoform of the intracellular enzyme which produces adenosine from adenosine monophosphate. As control, we used a vector tagged with GFP (Fig.6a). Two weeks after intravenous administration of viruses, an intense staining for cN-IA was observed in the liver of mice treated with cN-IA vector, particularly around lymphatic vessels (Fig.6b). A 2.5-fold increase in podoplanin-positive lymphatic vessels was detected in the liver of mice treated with cN-IA vector compared to mice treated with the GFP vector (Fig.6c). This increase was paralleled by enhanced expression of the pan-leukocyte marker CD45 (Fig.6d) and the macrophage marker F4/80 (Fig.6e). Therefore, over-expression of 5′-nucleotidase stimulates lymphangiogenesis and increases the expression of macrophage markers in the liver.

### Conditioned medium from macrophages increases the proliferation of LEC

**Figure 7:**
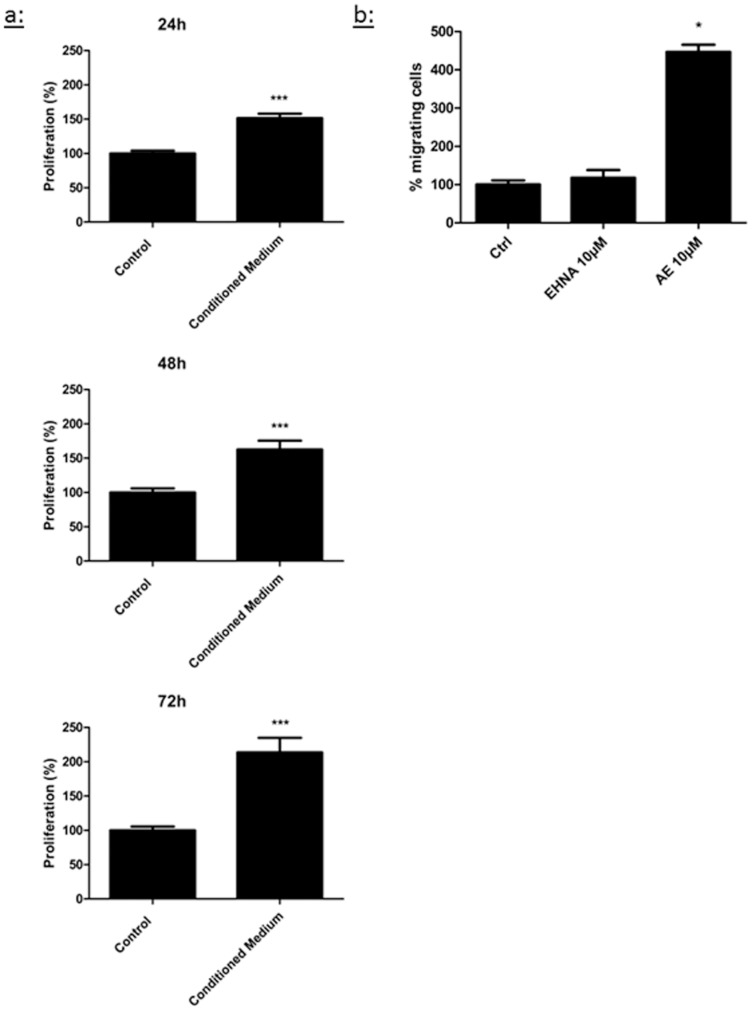
Effect of medium conditioned from macrophages on LEC proliferation and effect of adenosine on macrophage migration. (a)Proliferation of LEC was evaluated after 24 h, 48 h or 72 h in medium containing 2% FBS (Control) or medium conditioned by murine peritoneal macrophages obtained from naïve mice (Conditioned medium). Cell proliferation was measured with a CyQANT assay. (b) Migration of macrophages was evaluated using a Boyden chamber assay in which murine peritoneal macrophages obtained from naïve mice were incubated for 4 h with 2% FBS (Control) or adenosine with EHNA (10 μM each). Data are expressed as mean ± SEM (n = 3). * p<0.05 vs CTRL, *** p<0.001 vs CTRL.

To test a causal relationship between the presence of macrophages in the liver and enhanced lymphangiogenesis in mice injected with the adenovirus overexpressing 5′-nucleotidase, we exposed cultured LEC to conditioned medium from murine peritoneal macrophages obtained from naïve mice (i.e. not treated by the adenovirus). We observed a stimulation of the proliferation rate of LEC, reaching a 2-fold increase compared to control condition after 72 hours (Fig.7a).

### Adenosine stimulates the migration of macrophages

Finally, we addressed whether adenosine affects the migratory capability of macrophages. Using a Boyden chamber assay, we were able to determine that adenosine activates the migration of macrophages (Fig.7b).

## Discussion

The present study was designed to investigate whether adenosine modulates lymphangiogenesis. In vitro experiments showed that adenosine inhibits the proliferation and migration of cultured lymphatic endothelial cells. Most importantly, we observed that adenosine stimulates lymphangiogenesis in vivo, which might have therapeutic potential.

Since our initial hypothesis was that adenosine may directly bind to adenosine receptors present at the surface of LEC, we started our investigation with in vitro experiments involving cultured human primary LEC. We observed that adenosine consistently decreased cell proliferation and migration. This was not a consequence of cell death since adenosine was not cytotoxic for concentrations up to 10 μM. Adenosine had the optimal inhibitory effect at concentrations ranging from 1 to 10 μM, which are observed in the setting of pathological conditions such as ischemia. Indeed, physiological concentrations of adenosine are generally in the submicromolar range. Pharmacological in vitro studies with agonists of adenosine receptors suggested that the A2b receptor mediates the anti-proliferative effect of adenosine on LEC.

Surprisingly, we observed opposite effects in the whole animal, i.e. adenosine stimulated lymphangiogenesis. This observation was made in mice implanted with a gelatin sponge containing the stable analog of adenosine CADO, and in mice injected with an adenoviral vector encoding the sequence of the 5′-nucleotidase which results in over-production of adenosine [25], [26]. In mice implanted with gelatin sponges, we observed a blockade of the pro-lymphangiogenic effects of adenosine by MRS1754, an antagonist of adenosine A2b receptor, supporting the involvement of this sub-type of receptor in the effect of adenosine.

A possibility to explain the apparent discrepancy between in vitro and in vivo experiments may be the need for a microenvironment. Indeed, our data showing enhanced presence of macrophages in the liver where the adenovirus encoding 5′-nucleotidase accumulates suggest that adenosine stimulates lymphangiogenesis indirectly through macrophages. Accordingly, macrophages have been shown to drive lymphangiogenesis in different pathological conditions by secreting growth factors such as VEGF-C and VEGF-D [8], [9], [30], [33]. We [17] and others [31]–[36] have previously reported that adenosine is able to switch macrophages from an inflammatory M1 phenotype to a pro-angiogenic M2 phenotype associated with increased VEGF-A secretion and decreased inflammatory factors production. Although we failed to demonstrate that adenosine induces the production of VEGF-C by cultured macrophages (not shown), the possibility remains that adenosine may trigger the secretion of other pro-lymphangiogenic factors by macrophages.

The involvement of macrophages in the stimulation of lymphangiogenesis by adenosine is indirectly suggested by our present data showing that conditioned medium from macrophages, but not adenosine itself, stimulates LEC proliferation, and by the capacity of adenosine to enhance the migration of macrophages. Furthermore, a recent report by Keshet's group showed that VEGF induced the recruitment of circulating Ly6C^hi^ monocytes to the liver and endowed them with pro-angiogenic and pro-arteriogenic properties which stimulate vascularization [37]. To which extent infiltrated monocytes/macrophages, resident Kupffer cells and M-LEC contribute to the stimulation of lymphangiogenesis by adenosine is currently unknown.

A similar discrepancy in the in vitro and in vivo effects of adenosine in LEC observed in the present study is less expected to occur in vascular endothelial cells. Indeed, we previously reported that adenosine stimulates the production of VEGF-A by cultured macrophages [17] and it is known that adenosine has pro-angiogenic properties in vivo (unpublished data and [21] for review). Thus, adenosine directly and indirectly activates angiogenesis, which confers its role in tissue vascularization and repair.

Knowledge of the effects of adenosine on lymphangiogenesis, in addition to its effects on angiogenesis, finds its relevance in multiple biomedical fields such as oncology and any disease with an inflammatory component or a cellular stress. Indeed, under conditions of stress, production of adenosine is increased and adenosine receptors are activated [38], [39]. Tumor cells secrete adenosine in its environment [32] which can lead to 10- to 20-fold higher concentrations of adenosine compared to normal tissues [40]. Since lymphatic vessels participate in promotion of tumor growth and metastasis [3], it is tempting to speculate that blockade of adenosine receptors may result in decreased lymphangiogenesis in tumors, thereby participating to inhibition of tumor promotion [41]. While blocking antibodies against VEGFR-3 are currently tested as anti-angiogenic and anti-metastatic drugs [15], the potential for antagonists of adenosine receptors to inhibit lymphangiogenesis and its deleterious consequences on tumor development and anti-cancer therapies has not been addressed.

Finally, our findings may have some importance for non-cancer liver diseases since we have observed that adenosine stimulates lymphangiogenesis in the liver, and the lymphatic system is associated with several liver pathologies such as liver fibrosis, portal hypertension and cirrhotic ascites [42]. This is consistent with recent reports showing that antagonism of adenosine A2a receptor prevents and reverses liver fibrosis [43] and antagonism of adenosine A1 receptor reduces mortality of cirrhotic rats [44].

In conclusion, our study shows for the first time that adenosine stimulates lymphangiogenesis. This effect is dependent on the microenvironment and may involve macrophages. This observation may be useful for anti-cancer therapies, since adenosine is now recognized as a regulator of the complex interaction occurring between immune, inflammatory and endothelial cells [16].
